# Future Declines of Coronary Heart Disease Mortality in England and Wales Could Counter the Burden of Population Ageing

**DOI:** 10.1371/journal.pone.0099482

**Published:** 2014-06-11

**Authors:** Maria Guzman Castillo, Duncan O. S. Gillespie, Kirk Allen, Piotr Bandosz, Volker Schmid, Simon Capewell, Martin O’Flaherty

**Affiliations:** 1 Department of Public Health and Policy, University of Liverpool, Liverpool, United Kingdom; 2 Department of Statistics, Ludwig-Maximilians-University, Munich, Germany; Erasmus University Rotterdam, Netherlands

## Abstract

**Background:**

Coronary Heart Disease (CHD) remains a major cause of mortality in the United Kingdom. Yet predictions of future CHD mortality are potentially problematic due to population ageing and increase in obesity and diabetes. Here we explore future projections of CHD mortality in England & Wales under two contrasting future trend assumptions.

**Methods:**

In scenario A, we used the conventional counterfactual scenario that the last-observed CHD mortality rates from 2011 would persist unchanged to 2030. The future number of deaths was calculated by applying those rates to the 2012–2030 population estimates. In scenario B, we assumed that the recent falling trend in CHD mortality rates would continue. Using Lee-Carter and Bayesian Age Period Cohort (BAPC) models, we projected the linear trends up to 2030. We validate our methods using past data to predict mortality from 2002–2011. Then, we computed the error between observed and projected values.

**Results:**

In scenario A, assuming that 2011 mortality rates stayed constant by 2030, the number of CHD deaths would increase 62% or approximately 39,600 additional deaths. In scenario B, assuming recent declines continued, the BAPC model (the model with lowest error) suggests the number of deaths will decrease by 56%, representing approximately 36,200 fewer deaths by 2030.

**Conclusions:**

The decline in CHD mortality has been reasonably continuous since 1979, and there is little reason to believe it will soon halt. The commonly used assumption that mortality will remain constant from 2011 therefore appears slightly dubious. By contrast, using the BAPC model and assuming continuing mortality falls offers a more plausible prediction of future trends. Thus, despite population ageing, the number of CHD deaths might halve again between 2011 and 2030. This has implications for how the potential benefits of future cardiovascular strategies might best be calculated and presented.

## Introduction

The halving of UK Coronary Heart Disease (CHD) death rates (mortality) since the 1970s represents a major public health achievement. However, CHD still remains the leading cause of premature death (before 75 years) and years of life lost (YLLs) in the UK [1]. Strategies to maximise the prevention of CHD and cardiovascular disease (CVD) therefore remain a top policy priority. The English government recently suggested that the NHS Health Checks programme might prevent or postpone approximately 650 cardiovascular deaths per annum [2].

From the late 1980s, scientists started to recognise early signs of a deceleration in previous CHD mortality declines. In the United States, the annual rate of decline for men aged 35 to 54 fell from 6.2% per annum in the 1980s to 0.5% in the 2000s [3]. Similar warning trends were discovered in other countries: England and Wales [4], Scotland [5], the Netherlands [6] and Australia [7]. In England and Wales, CHD mortality declines were not just slowing [4] but in 2002 for the first time in over two decades, CHD mortality increased among men aged 35–44 years.

CHD mortality rises approximately exponentially with age. Thus the population ageing now occurring in most industrialised countries could drive a higher CHD burden in future years. This shift in population density to older ages has been driven by continued reductions in mortality, and the influx of birth cohorts from the first and second baby boom generations, who are now approaching retirement age [8]. It is therefore not surprising that by 2030, the largest predicted shifts in age distribution are anticipated in the older groups aged 65 and beyond: 35% among those 65–74 years old, 50% among those aged 75–84, and 89% in those aged 85 and beyond. This is truly a dramatic shift in population density to older ages.

The result will be a rapid and sustained rise in numbers of UK patients requiring care for both morbid and fatal CHD events. In the US, Heidenreich et al. [9] predicted that around 40.5% of the population will have some form of CVD by 2030. In England and Wales, the only certainty is that the increasing burden of CHD will place a huge strain on the National Health Service and on informal carers [10].

Forecasting mortality (all-cause and disease-specific) is the art and science of demographers, actuaries, epidemiologists and policy makers. The range of methods includes parameterisation models (i.e. mortality laws), the widely applied Lee-Carter method, age period cohort models, statistical models for mortality and morbidity and dynamic multi-state models (see Booth and Tickle [11] and Tabeau et al. [12] for a review of methods).

There have been several forecasts of the potential future of CVD burden: Heidenreich et al. [9], Huovinen et al. [13], Odden et al. [14], Huffman et al. [15], Heidenreich et al. [16] and Ovbiagele et al. [17]. However, these forecasts have used the assumption that any current decline in CVD morbidity and mortality will cease at the beginning of the forecast period. In other words, the prevalence and mortality rates are held constant when projecting numbers of cases and deaths into the future. The International Diabetes Federation also assumes constant rates to predict future global burden of the disease [18].

Perhaps of more concern is the evidence that policy makers use similar approaches. For example, the English NHS Health Checks programme is based on the assumption that the risk of dying from CVD will persist unchanged in the future [19]. This sudden cessation of mortality decline would represent a catastrophic (and hopefully unlikely) deterioration of UK public health.

Therefore the aim of this paper was to contrast the predictions made under the assumption of a sudden cessation of mortality decline in England and Wales with predictions made under the assumption of continuation of recent declines.

## Methods

We obtained data on the number of CHD deaths from 1979–2011, stratified by gender and 10-year age groups (35 to older than 85 years) in England and Wales, from the Office of National Statistics (ONS). CHD is defined as codes 410–414 and I20–I25 in the 9^th^ and 10^th^ versions respectively, of the International Classification of Diseases. Mid-year population estimates were obtained from the same source along with 2010-based population projections for the period 2012–2030. From these data, we computed mortality as the central deaths rate from CHD.

### Scenarios

In scenario A we assumed a conventional counterfactual, that the last-observed levels of CHD mortality (in 2011) would persist unchanged. To obtain indirect standardised estimates of the future number of CHD deaths, we multiplied this CHD mortality by the forecast population numbers.

In scenario B we assumed that the recent trends in CHD mortality rates might continue. We evaluated two conventional models to forecast CHD mortality rates from 1979 to 2011: the Lee-Carter model [20] and the hierarchical Bayesian Age Period Cohort (BAPC) model [21]. To assess the prediction performance of both models, we used data from 1979 to 2001 to predict mortality rates from 2002 to 2011. Then, we computed the mean absolute percent error (MAPE, see Text S2 in Appendix S1) between those projections and the observed rates from the same period of time. The model with the lowest MAPE was used to forecast the number of CHD deaths (using the population projections until 2030).

We then investigate the difference between the forecasts from scenario A and the best model from scenario B. We show this in terms of forecast CHD mortality rates and number of CHD deaths.

Finally, we assessed the prediction performance of scenario A and scenario B using MAPE, in the same manner as when comparing the Lee-Carter and BAPC models.

#### Lee-Carter and BAPC models

Lee and Carter [20] developed a method to forecast mortality that combines a demographic model with time-series methods of forecasting. The basic idea behind the model is that a single parameter (mortality index) governs the dynamics of a mortality trend. It is the most widely-used method for forecasting future mortality and numerous extensions have been developed in the last decades.

Let 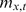 be the central mortality rate for age 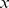 in year 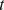
_
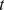
_, the model states
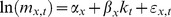
Where 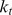 is the mortality index at time 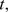 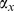 is the average pattern of mortality by age, 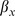
is the relative change with respect to the mortality index at age 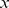 and 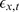 is the residual at age 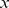 and year 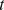.

We fitted the basic Lee-Carter model to our data using the R package demography [22]. Because we have data available only in 10-year age-groups, prior to fitting the model, we used monotonic regression splines for smoothing [23].

Because of it is nature, Lee-Carter models are more suitable for forecasting mortality trends characterised by a strong period component effect, such as infectious respiratory diseases [24]. Period effects might capture temporal change in factors associated with medical and broader societal development. However, we expect that (albeit potentially small) cohort effects are also important to the aetiology of CHD. Cohort effects capture generational differences temporal change in life-course factors by year of birth. The BAPC model allows investigating the additional cohort effects in mortality along with age and period effects.

The BAPC model assumes the logit of the mortality probability from CHD in age group *i* in period *j* is a linear combination of an intercept 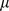, age effects 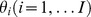, period effects 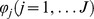 and cohort effects 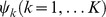:
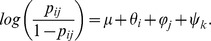


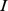
 is the total number of age groups and 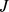 the total number of periods. Cohorts are defined by 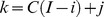 and the total number of cohorts is 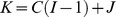, where 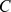 is the width of the age bands. In our data, *I* = 6, *C* = 10 and *J* = 33, hence there are *K* = 83 cohort parameters.

We estimated the age, period and cohort effects between 1979 and 2011 and then let each of these components continue along their last observed linear trends. Then we extrapolated the resulting trajectory to 2030. We also re-estimated the model by adding a parameter that estimates unobserved heterogeneity in the input data not explained by age, period and cohort effects.

The models were implemented in the Bayesian Age-Period-Cohort Modeling and Prediction software (BAMP) [25] which uses Markov Chain Monte Carlo (MCMC) for the model estimation. We evaluated the goodness of fit of the models with and without the heterogeneity parameter using the Deviation Information Criterion (DIC) [26] which also penalises for model complexity.

Finally, to assess the importance of the cohort (along with age and period) effects for CHD mortality, we re-estimated the model by relaxing some assumptions imposed to the parameters (see Text S1C in Appendix S1). For more details about the BAPC methodology, please see Text S1 in Appendix S1. For more details about the software, please see Schmid and Held [25].

## Results

### Lee-Carter vs BAPC Model

According to the DIC values (Table 1), the best fitting BAPC model was the model with the heterogeneity parameter. Smaller DICs indicate a better supported, more parsimonious model. The strong support for a heterogeneity parameter indicates that there was still substantial unexplained variation around our main model fit.

**Table 1:** DIC values for BAPC models with and without heterogeinity parameter.

Model	Men	Women
BAPC	1122	1438
BAPC + heterogeneity	381	349

Smaller DICs indicate a better supported more parsimonious model. See Text S1B in Appendix S1 for details of the model fits.

**Figure 1:**
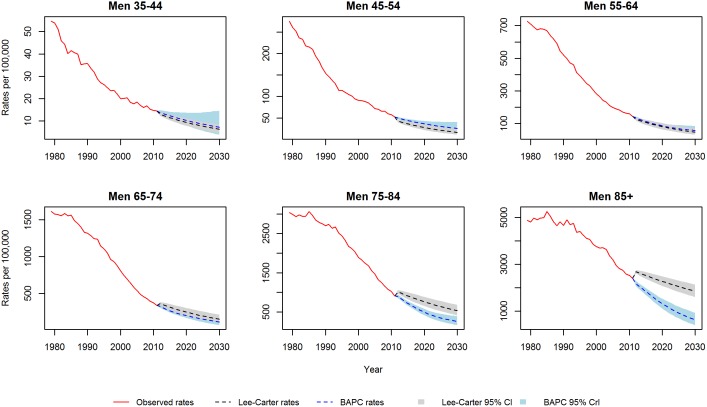
CHD mortality rates for men: Observed rates, Lee-Carter and BAPC projections.

**Figure 2:**
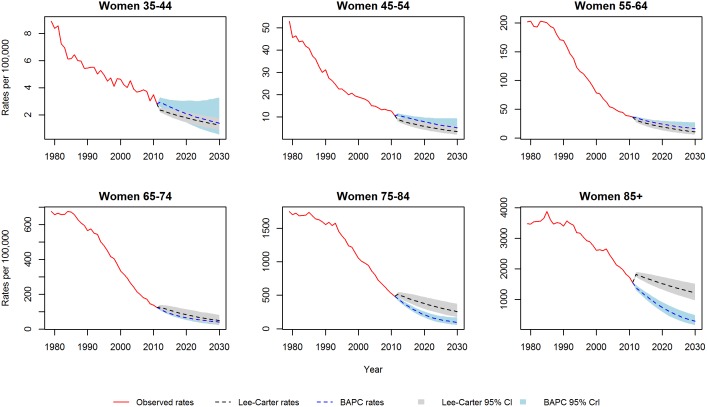
CHD mortality rates for women: Observed rates, Lee-Carter and BAPC projections.

Figures 1 and 2 show the CHD mortality rates estimated by the Lee-Carter and BAPC models. Both models suggest mortality rates, for each gender and at all ages, will follow a continuing but decelerating trajectory of decline to 2030. In particular, for the middle age groups (45–54, 55–64 and 65–74) both models predict a substantial slowing of the decline. Additionally, the Lee-Carter differs from the BAPC model in predicting a slower decline at the eldest ages.

**Figure 3:**
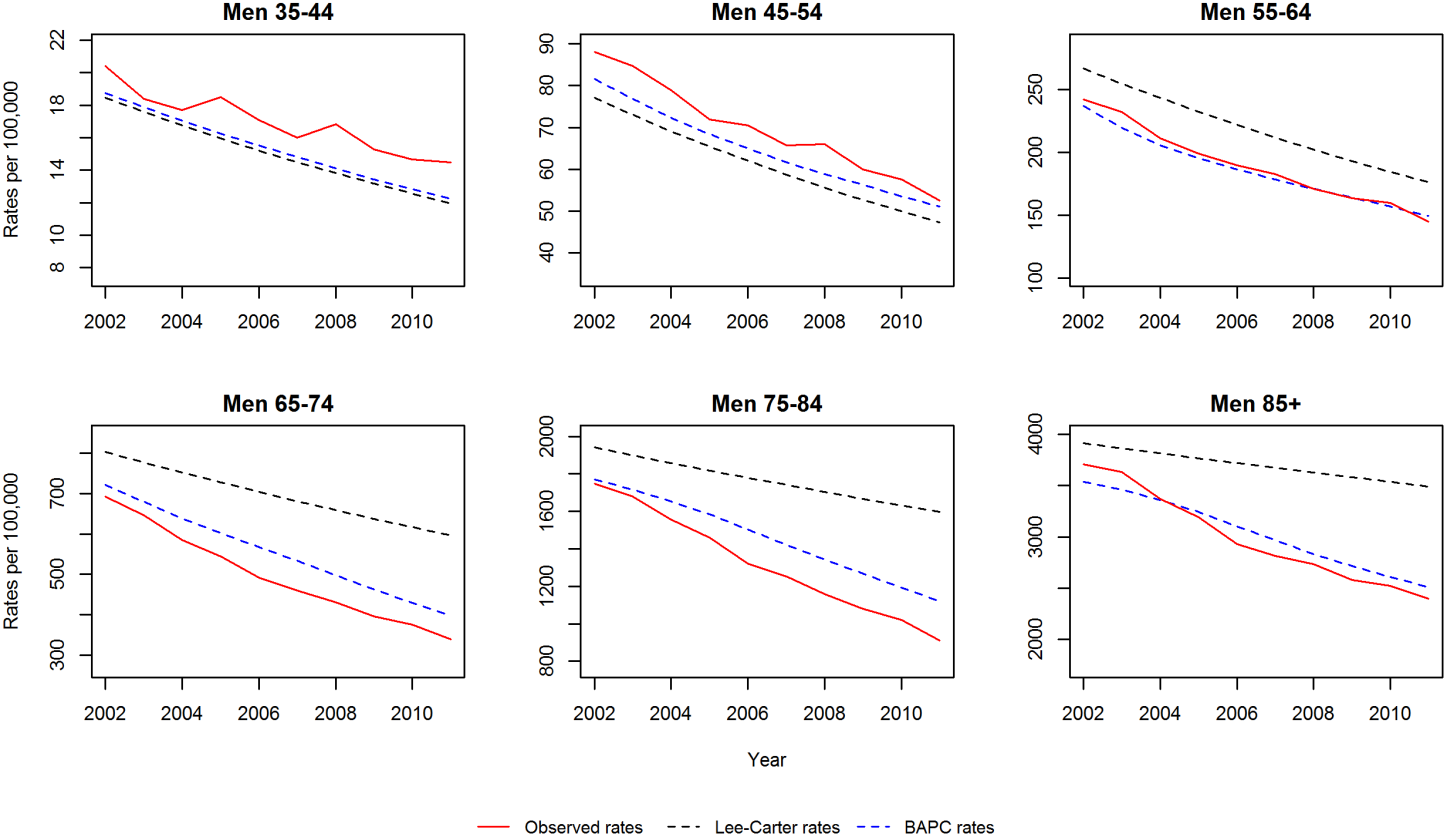
Validation of mortality rates for men 2002–2011: Observed rates, Lee-Carter and BAPC projections.

**Figure 4:**
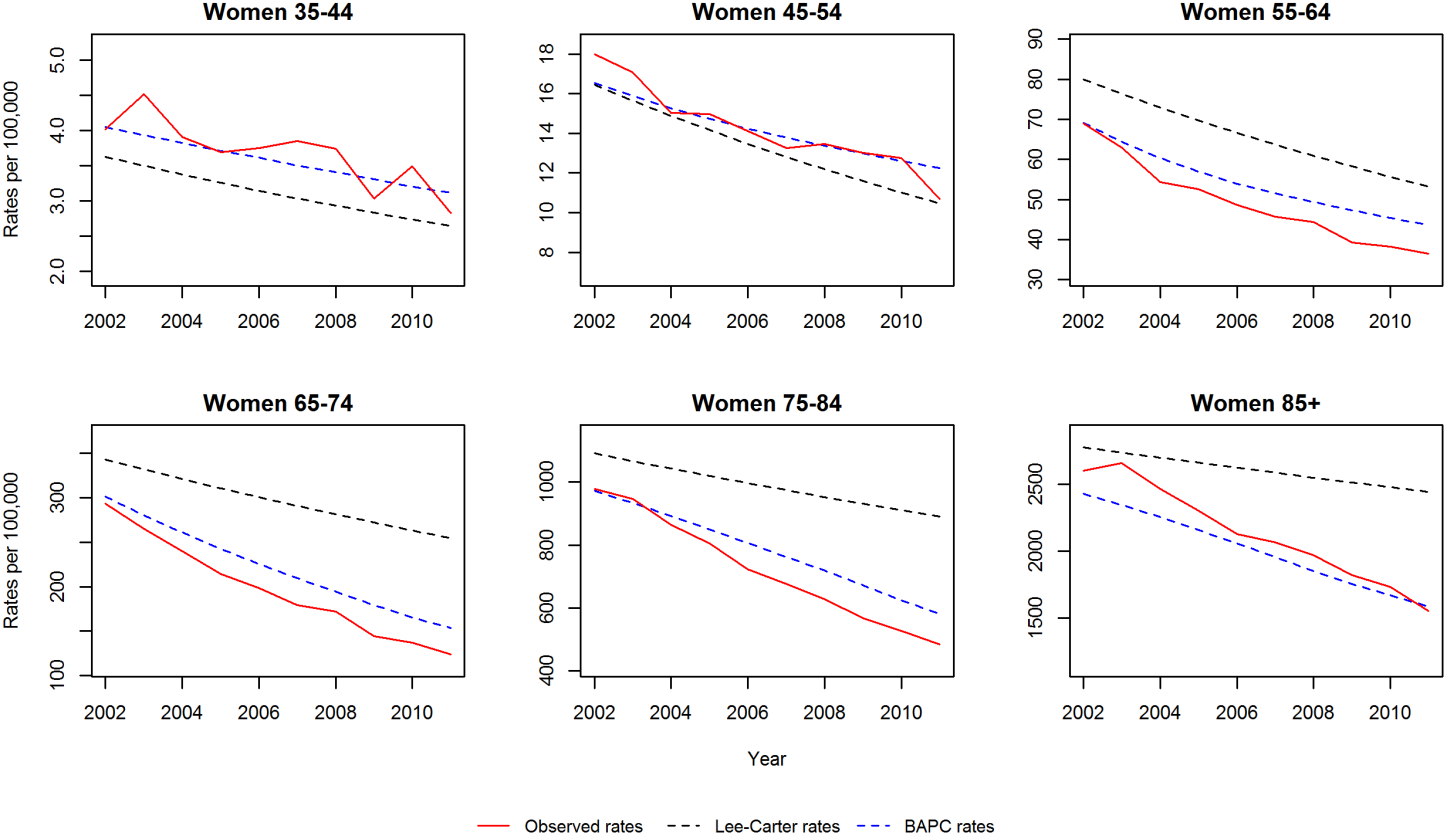
Validation of mortality rates for men 2002–2011: Observed rates, Lee-Carter and BAPC projections.

**Figure 5:**
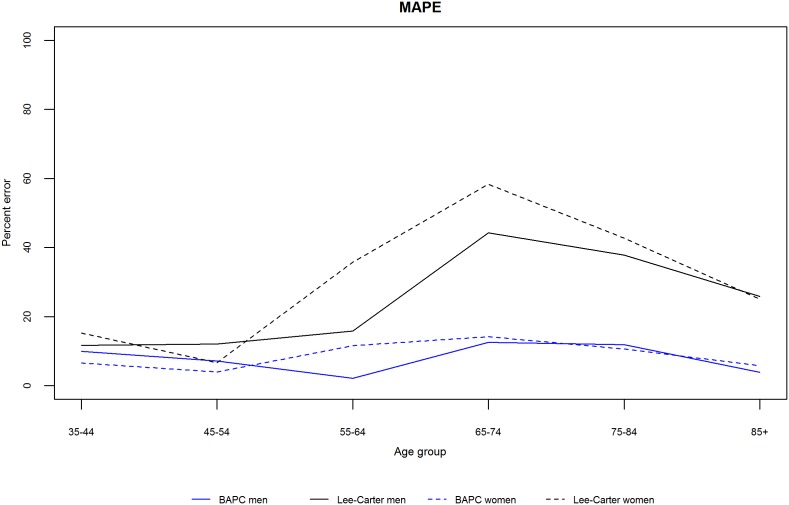
Mean absolute percent error between predicted (Lee-Carter and BAPC) and observed rates by age group and gender.

We initially validated each model by projecting mortality from 2002–2012 based on data from 1979–2001, and subsequently compared our projections with the observed data. Figures 3 and 4 show the projected and observed values. The BAPC model provides a very good fit, although for age groups 65–74 and 75–84 in men and women, it predicted slower declines than observed. The Lee-Carter model highly overestimated those above 55 years of age. In terms of MAPE (see Figure 5), The BAPC model had a lower percentage error than the Lee-Carter model. Thus, although both models agreed mortality will continue to decline, we choose the BAPC model to continue our analysis as it had better predictive performance.

#### Interpretation of the age, period and cohort effects

To estimate the age, period and cohort effects from the BAPC model, we need to relax the restrictions imposed to the model (see Text S1C in Appendix S1). The results suggest that the age effect explained most variation; followed by the period effect, and then the cohort effect.

### Constant Future Mortality Versus Continuing Declines

#### Mortality rates

**Figure 6:**
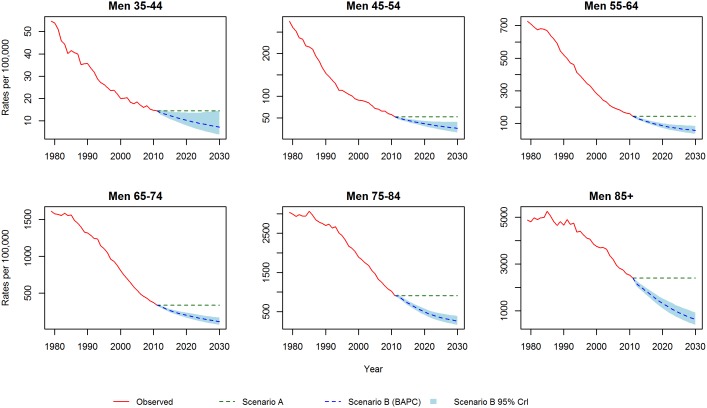
CHD mortality rates for men: Scenario A and Scenario B.

**Figure 7:**
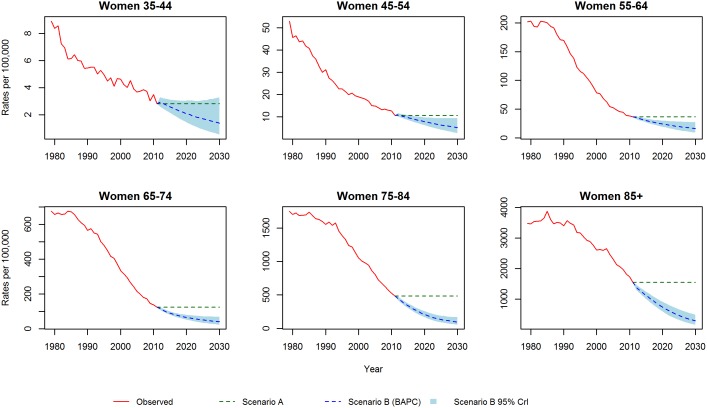
CHD mortality rates for women: Scenario A and Scenario B.

Figures 6 and 7 show the projected CHD mortality rates under scenario A (constant 2011 levels of CHD mortality) and scenario B (the BAPC model). In scenario B, mortality rates for each gender and at all ages follow a continuing but decelerating, trajectory of decline to 2030. The expected rate of future decline is notably faster at ages 75–84 and older, especially in males: over age 85, future CHD mortality could fall by a difference of 1,270 deaths per 100,000 individuals [95% credible interval (95% CrI) 1,065–1,386] in females, and by 1,770 deaths per 100,000 individuals (95% CrI 1,470–2,000) in males. However, for the middle age groups (45–54, 55–64 and 65–74) the BAPC model in scenario B predicts a clear slowing of mortality decline.

#### Number of deaths

**Figure 8:**
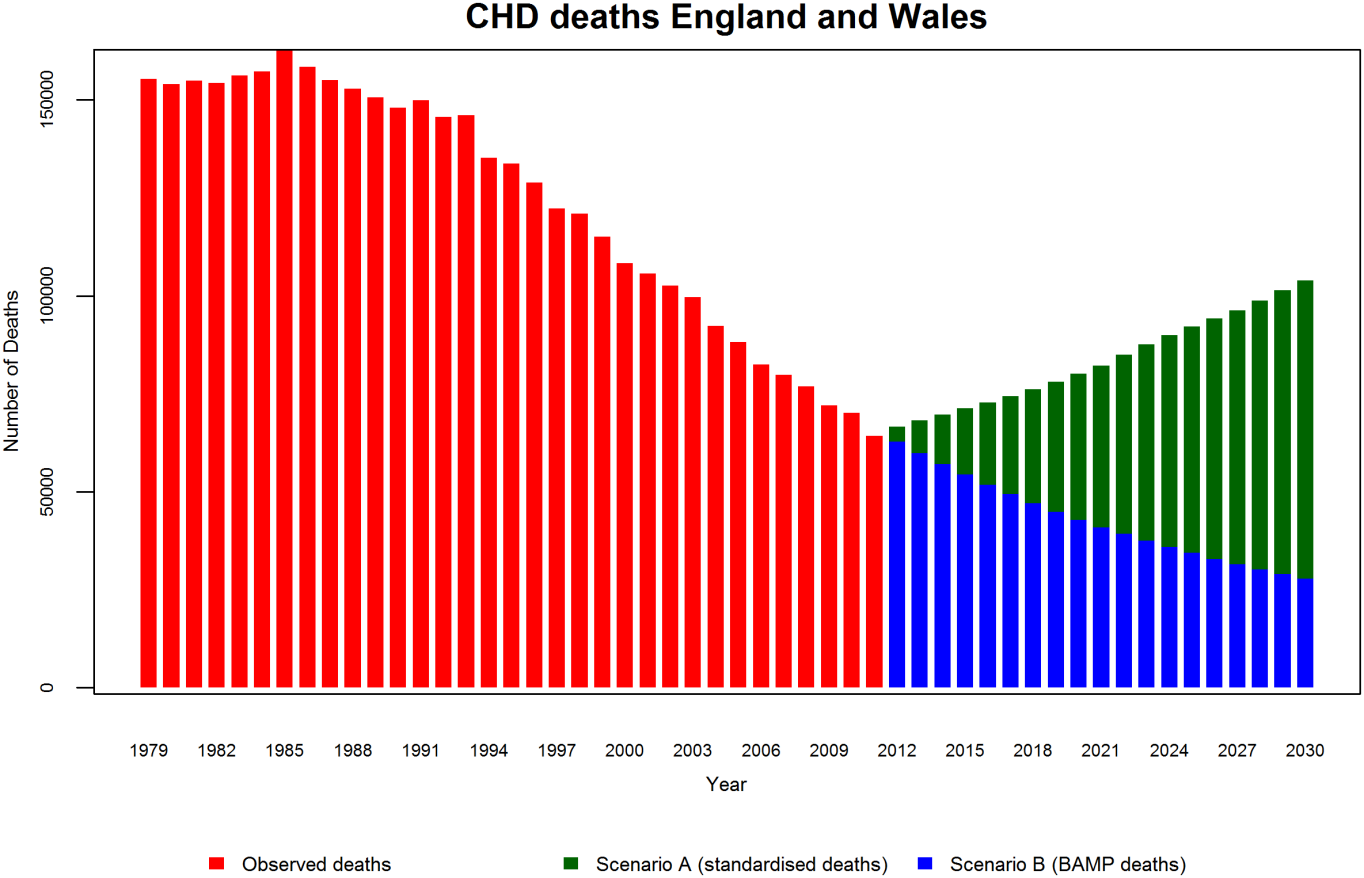
Total number of CHD deaths 1979–2030.

If CHD mortality remains at its 2011 level (scenario A), by 2030 we can expect that the total number of CHD deaths will increase substantially by 67% [95% confidence interval (95% CI) 65%–68%] for men and 54% (52%–55%) for women; with an overall increase of 62% (61%–63%) or 39,600 deaths. This is due to population ageing, which increases the number of people present at older ages, thus generating more CHD deaths. However, if recent declines in CHD mortality continue (scenario B), the BAPC model suggested that absolute number of deaths would decrease by 49% (95% CrI 24%–68%) for men and by 66% (43%–81%) for women, yielding a total decrease of 56% (29%–74%) or 36,200 deaths. Figure 8 shows the total observed numbers of CHD deaths and projections under both scenarios. We can observe that the number of deaths has fallen rapidly since the 1980s. If CHD mortality is assumed to stay constant at 2011 levels then the past trend reverses abruptly, to a rapid increase in CHD deaths. In stark contrast, the BAPC projects a continuing decline, albeit at a decelerating rate.

**Figure 9:**
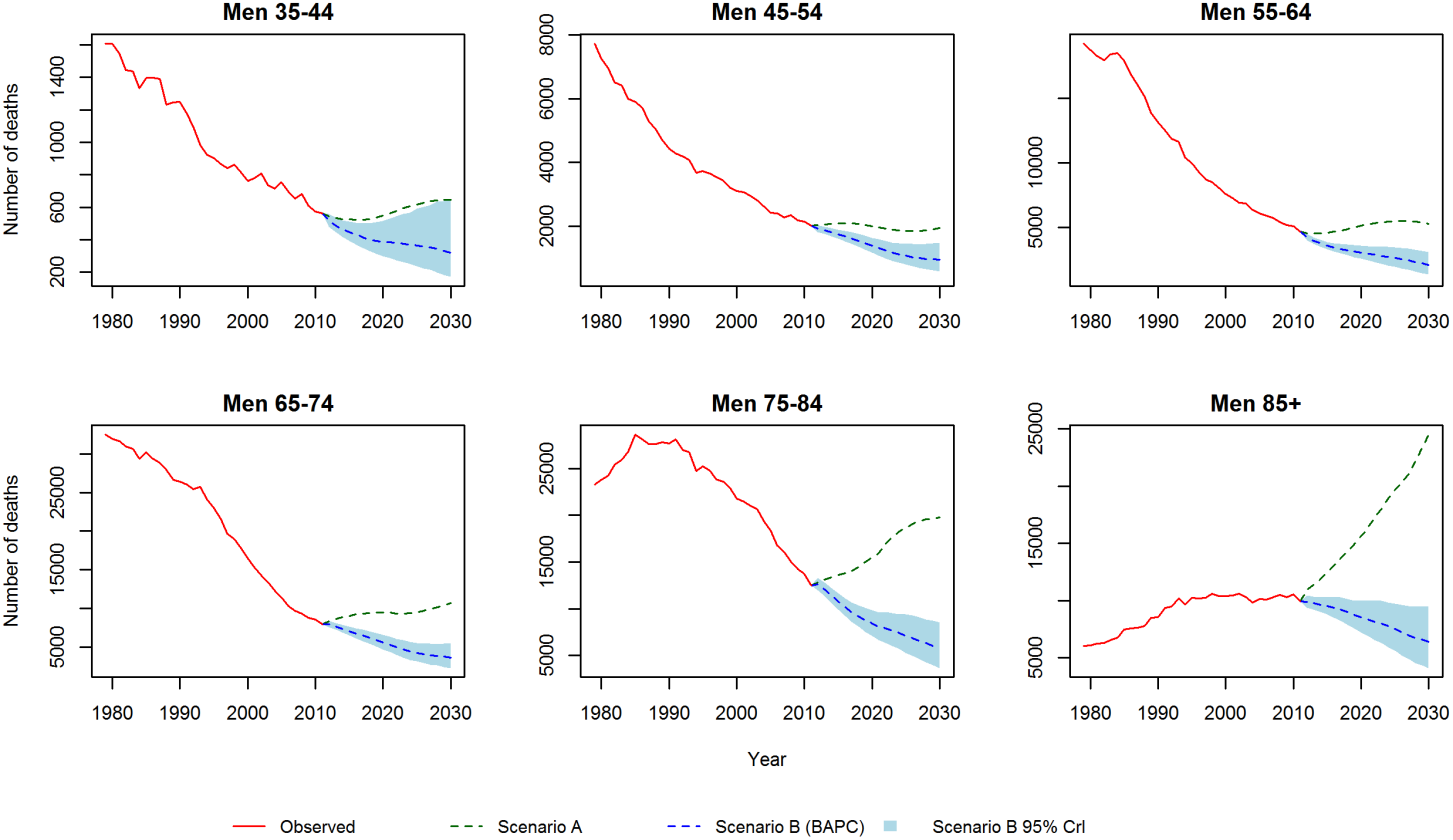
CHD deaths for men: Scenario A and Scenario B.

**Figure 10:**
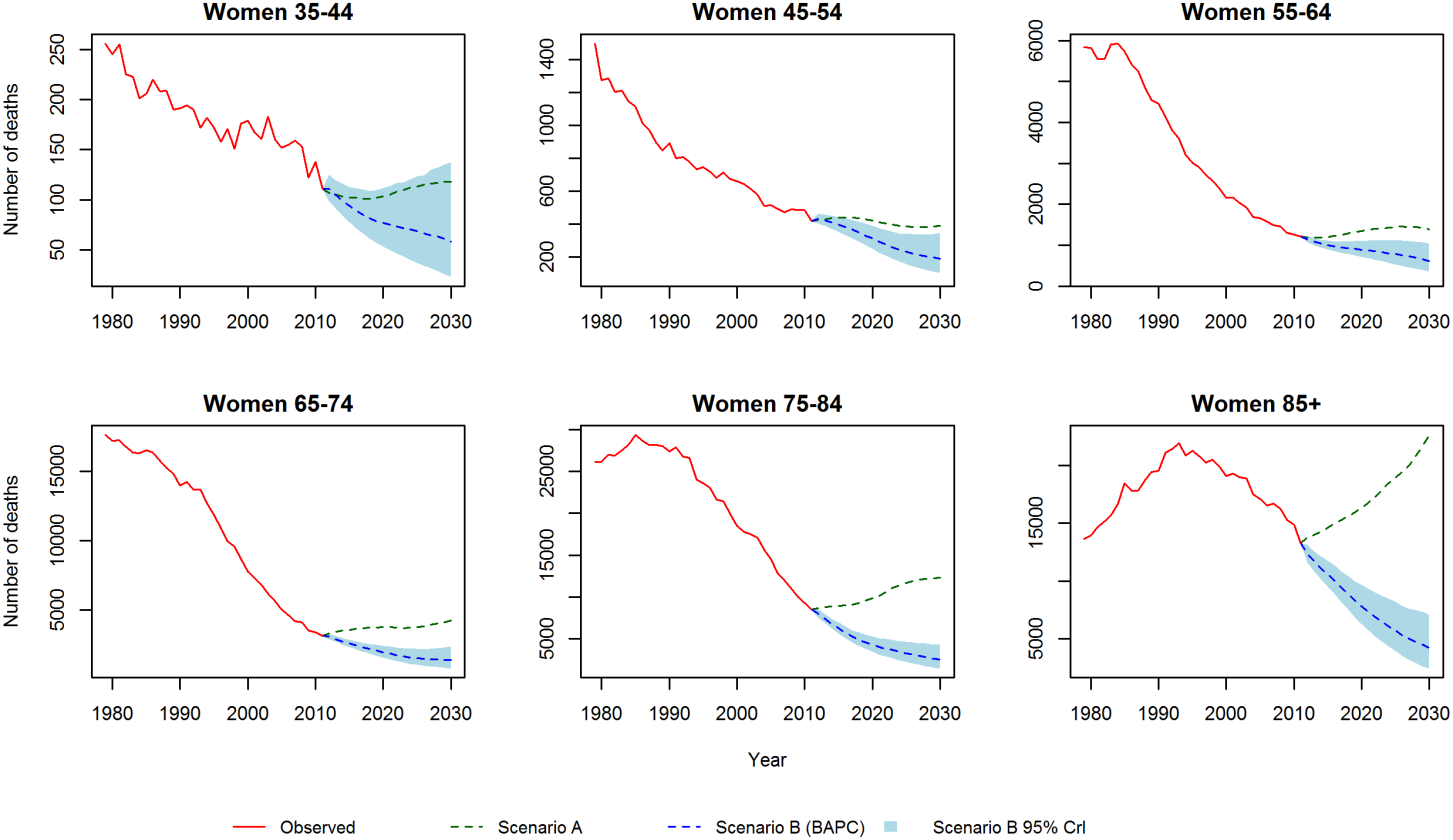
CHD deaths for women: Scenario A and Scenario B.

These results are consistent with the projections stratified by age group and gender (Figures 9 and 10). In scenario B, we no longer see pronounced decline in deaths among men and women as evident as in the mortality rates, due to the future influx of people into the oldest age classes (>75 years) which slows the decline in number of deaths. Similarly, we observe a clear slowing in the decline of absolute numbers of deaths for middle groups 45–54, 55–64 and 65–74.

It is striking how opposite the trends with scenario A are to the natural trend observed in the last 30 years. The BAPC model projections continue the past trends and lead to a powerful conclusion: The projected CHD mortality declines are more than adequate to counteract the wave of additional CHD deaths that would be generated by population ageing.

**Figure 11:**
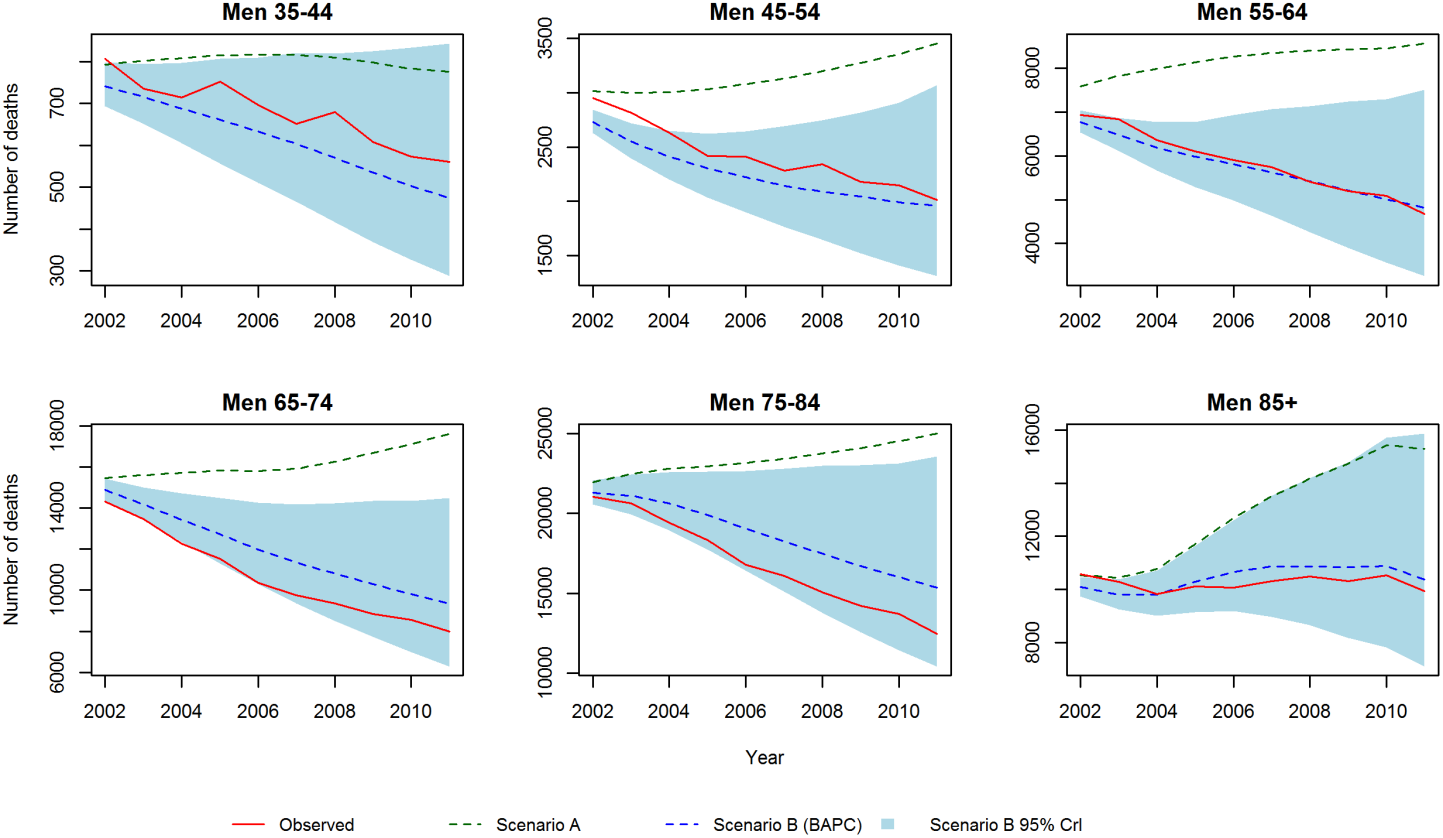
Validation of mortality rates for men 2002–2011: Observed rates, Scenario A and Scenario B.

**Figure 12:**
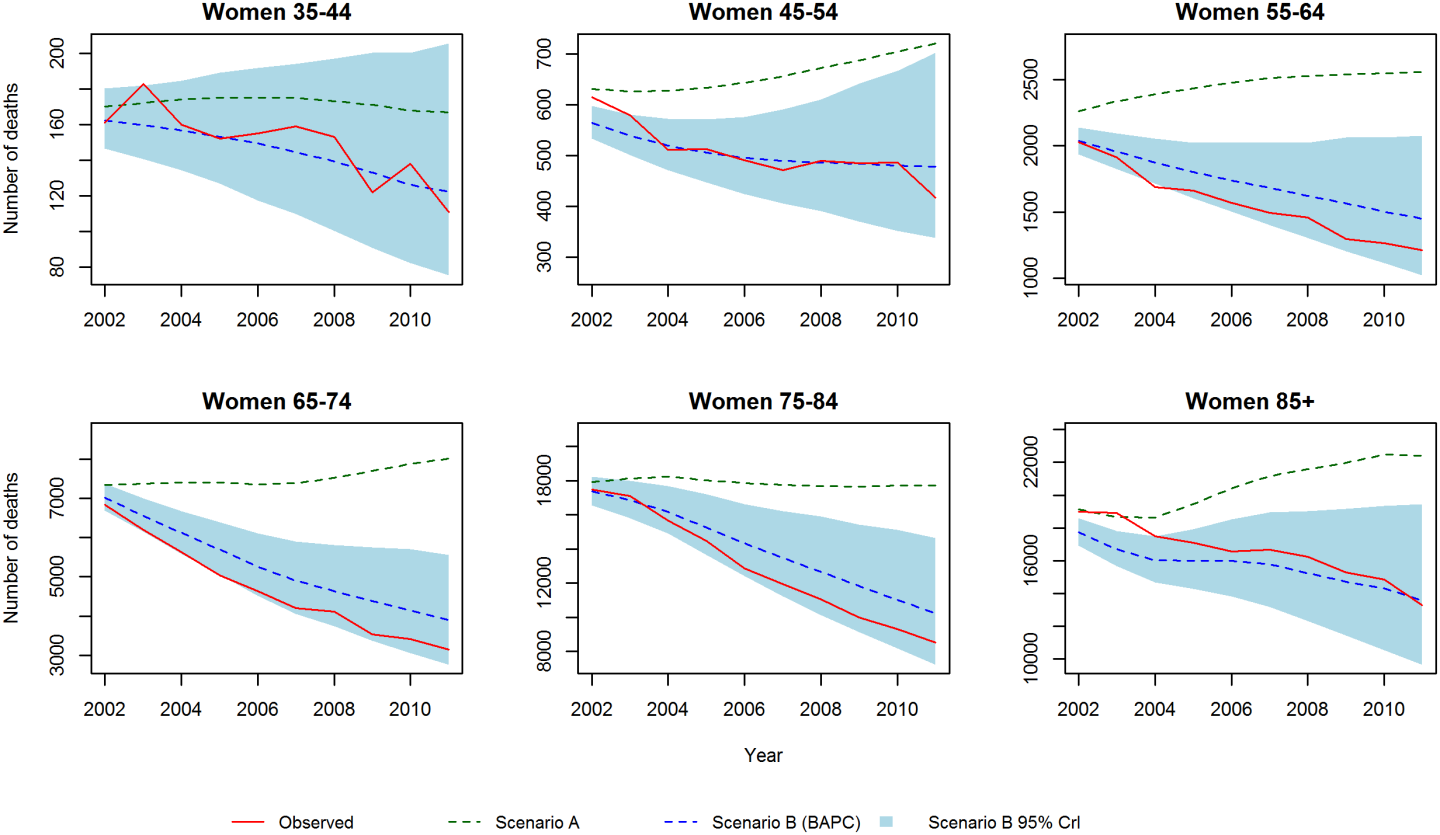
Validation of mortality rates for women 2002–2011: Observed rates, Scenario A and Scenario B.

**Figure 13:**
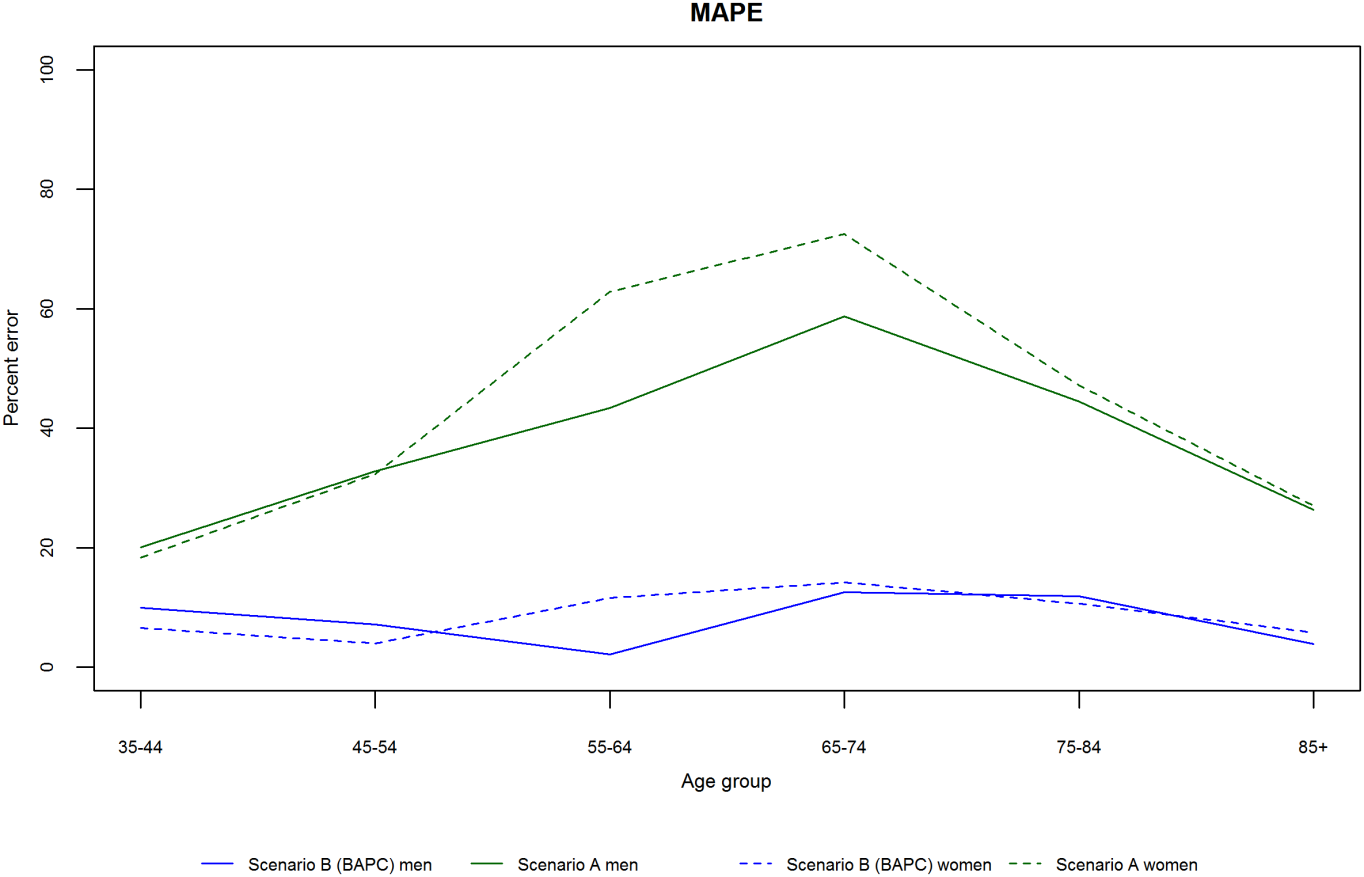
Mean absolute percent error between predicted (Scenario A and B) and observed rates by age group and gender.

Finally to assess the prediction performance of both scenarios, we estimated the number of deaths from 2002–2012 and compared these with the actual observed values. For scenario A, we projected mortality rates at 2001. For scenario B, we used data from 1979–2001 to re-estimate the model. Figures 11 and 12 show the predictions and observed values. Scenario A highly overestimated the number of deaths, whereas scenario B provides a reasonable fit. In terms of MAPE (see Figure 13), scenario A had a consistently higher percentage error than scenario B.

## Discussion

### Main Findings

We evaluated two scenarios in relation to future estimates of CHD deaths. In scenario A, we assumed that the mortality rates in 2011 would persist and remain unchanged until 2030. Under this very conservative assumption, we estimated that the total numbers of CHD deaths would increase substantially by 67% for men and by 54% for women. These predicted rises reflect the demographic changes that England and Wales will experience in the next decades, notably population ageing. However, in scenario B by simply assuming that the recent declines in CHD mortality might continue, our estimates of the future burden of CHD mortality are substantially lower (−49% for men and −66% for women).

### Lee-Carter vs BAPC

We compared two commonly used methods to forecast mortality: The Lee-Carter model and the Bayesian estimation of an APC model. Because we found a very small cohort effect in the CHD mortality trends, it was expected that both models would give similar results. Indeed both models projected declines in mortality up to 2030.

However, when their prediction performance was evaluated, Lee-Carter seemed to perform poorly for the eldest groups. Why Lee-Carter tends to underestimate mortality decline in the eldest groups may be because the ratio of rates of proportional mortality change (i.e. the change in log mortality) at different ages is assumed to remain constant over time [11]. Therefore Lee-Carter is relatively insensitive to trends in the age-pattern of mortality change. Indeed, as Darkiewicz and Hoedemakers [27] shown for all-cause mortality data for England and Wales, it is not unusual for the age-pattern of mortality change to vary over time. These authors also fitted a Lee-Carter model and found that the model seriously under-estimated mortality decline for elderly men and women. This issue with Lee-Carter has been thoroughly discussed and methods to deal with it has been suggested by Booth et al. [28] and Carter and Prskawetz [29] among others. Thus, our use of the BAPC model also seems advantageous on statistical grounds.

### Comparison with other Studies

Many of the previous studies assuming constant mortality rates (scenario A) also reported projected estimates of the number of future CHD deaths. For example, the Coronary Heart Disease Policy Model is a complex Markov model that simulates the future incidence, prevalence and mortality from CVD in the U.S [14]. Their results suggested that if no significant changes in risk factors or treatment occur in the future, the annual number of CHD deaths will increase by 56% over the next 30 years. Similarly, Huovinen et al. [13] predicted an increase in deaths among Finnish men and women aged 60 and older, including an alarming 202% increase in deaths among men aged 80 and older. Also, Huffman et al. [15] reported that if risk factor trends and mortality rates in the U.S. population remain constant (at the 2006 level), there could be an increase of 12% in absolute numbers of deaths by 2020.

However, as we have demonstrated, the assumption of mortality remaining constant appears neither realistic nor valid. The consequent predictive error can be vast, for example, an early version of the Coronary Heart Disease Policy Model (1987) predicted that by 2010 the total number of CHD deaths in the US would be around 630,000 [30]. However, the observed value (380,000 deaths, [31]) in 2010 was substantially lower, barely half their prediction.

In contrast, our scenario B assumes (not unreasonably) that recent mortality trends may continue for some time into the future. Under this more plausible scenario, we suggest that the total number of deaths in England and Wales in 2030 could decrease substantially by 47% in men and by 66% in women.

Huovinen et al. [13] used a similar BAPC analysis to predict CHD mortality in Finland. Their results are very similar to ours, except for the oldest group: They predicted that the number of deaths among men aged 80 and older would increase more than four-fold between 2002 and 2030, and almost three-fold among women. Although this could reflect population differences between the UK and Finland, it might also reflect differences in the model assumptions: Huovinen held the APC components constant over the forecast period whereas we allowed their trends to continue (see Text S1A in Appendix S1). In addition, the CrIs for the oldest group are particularly wide, highlighting the difficulty of reliable predictions for the elderly. For example, for men 80 and older, Huovinen’s 95% CrIs range between 474 and 2,620 deaths.

### Cohort Effects

Another interesting result from our scenario B (BAPC model) is that the most important factor for CHD mortality in both sexes is age, followed by period and cohort effects. Cohort has a very small effect. There is contradictory evidence regarding whether there are detectable cohort effects on CHD mortality. APC models built for Australia [32] and New Zealand [33], and other descriptive analyses carried out for Poland and Hungary [34], suggested the absence of any cohort effect. In contrast, modest cohort effects in CHD mortality were reported in Singapore [35] and Norway [36], and a descriptive study in Hong Kong [37] found cohort effects for women but not for men. Such conflicting evidence may be partially attributable to the fact that most of the studies claim cohort effects are caused by events that operate during the perinatal period [38]. On the other hand, the major changes in cardiovascular risk factors have been period-based changes to lifestyle and treatments [32]. Cohort effects can also be a consequence of influences later in life. For example, social attitudes regarding lifestyle could be the result of new cohorts with different risk behaviours replacing older cohorts [39].

### Strengths and Weaknesses

The BAPC model offers some further important advantages: the classical APC model allows mortality to be described as trends over age at death, year of death and birth cohort, and to model extra heterogeneity if it is needed. Likewise, the Bayesian setting exhibits some interesting features that increase the reliability and interpretability of our predictions: Prior information (e.g. results of a previous model or expert opinion) can be used to guide the inference made from the current data. The Bayesian approach estimates the probability distribution of the parameters, given the data. This yields a full probability model that, unlike frequentist methods, can be used to generate a probability distribution of possible futures. This probabilistic distribution of futures can be summarised by 95% credible intervals, which are probabilistic regions around the estimates and differs from frequentist confidence intervals by allowing direct comparison among the probabilities of possible futures.

In addition, Bayesian models are unbiased with respect to the sample size; therefore, the model works well with smaller numbers of cases [40], which is useful when modelling women and younger groups.

However, our projections also have limitations that should be acknowledged. First, the projections are calculated using future estimates of population (produced by the ONS). These population projections are based on a number of assumptions regarding future levels of fertility, migration and mortality. All can present difficult tasks for demographers and statisticians [41] and the estimates produced may be biased. However, one possible solution would be to fit BAPC models separately for different levels of migration.

Second, our projections assume that effects of the age, period and cohort components will remain unchanged into the future. However, CHD mortality can sometimes change dramatically over a short period of time. These “black swan” events are undirected and retrospectively unpredictable [42]. For example, following the break-up of the Soviet Union in 1989, profound changes in diet in Poland and neighbouring countries were associated with sudden large decreases in CVD deaths commencing in 1990 [43], [44]. Equally rapid CVD mortality falls were observed in Cuba after a sharp and substantial reduction in calorie intake during their so called “special period” of the early 1990’s. The lag times observed were consistently less than five years [45], [46]. Even more rapid rises and falls in CVD mortality have recently been observed in Russia, partly reflecting the dramatic fluctuations in consumption of alcohol [47], [48]. Similarly, rapid decreases in mortality have been seen after smoke-free legislation in Scotland and in other populations [49].

In contrast, CHD rates can demonstrate a lag time of one or two decades following increases in obesity or diabetes [50]. The continuing dramatic rises in obesity and diabetes prevalence in the UK, coupled with recent flattening in blood pressure and cholesterol trends in the last decade, make predictions about the future evolution of the CHD epidemic extremely uncertain [51]–[54]. Even assuming constant mortality rates (our scenario A), Huffman et al. demonstrated that, simply by considering plausible changes in the risk factors trends, their results varied by 27% from their baseline (no-changes) scenario [15].

### Conclusions and Policy Implications

The global public health community is increasingly focussing on strategies to control non-communicable diseases [55]. Thus, past and current trends in risk factors and mortality might give health service planners useful insights on the evolution of the CHD epidemic. However, we will need disease burden forecast methodologies which exploit the increasingly detailed causal knowledge of CVD. Department of Health modelling has suggested that the English NHS Health Checks programme might prevent or postpone approximately 650 cardiovascular deaths per annum. However, that figure might represent a serious over-estimate, because it assumes that the risk of dying from CVD will persist unchanged in the future [19]. If so, this makes the estimated annual cost of NHS Health Checks of approximately £300 million look even more substantial.

## Supporting Information

Appendix S1 **Supplementary information.** Text S1, Bayesian age, period and cohort model. Detailed description of the BAPC models. Text S1A, Random walk of first and second order. Description of the different type of parameters assumptions for the age, period and cohort effects. Text S1B, Estimation, prediction and comparison. Description of the methods to estimate BAPC models, how to compare between different models and how to compute projections into the future. Text S1C, BAPC model for CHD mortality in England and Wales. Specific methods and assumptions used for the CHD mortality BAPC model in England and Wales. Text S2, Mean absolute percent error. Description of the type of error measurement used to compare scenarios and models. Text S3, References. References used in the Supplementary information section.
(DOCX)
