# Supplementary material for: Future Declines of Coronary Heart Disease Mortality in England and Wales Could Counter the Burden of Population Ageing
Source: PLoS One. 2014 Jun 11;9(6):e99482. doi: 10.1371/journal.pone.0099482 (PMC4053422; doi:10.1371/journal.pone.0099482)
Supplement: Appendix S1 — Supplementary information. Text S1, Bayesian age, period and cohort model. Detailed description of the BAPC models. Text S1A, Random walk of first and second order. Description of the different type of parameters assumptions for the age, period and cohort effects. Text S1B, Estimation, prediction and comparison. Description of the methods to estimate BAPC models, how to compare between different models and how to compute projections into the future. Text S1C, BAPC model for CHD mortality in England and Wales. Specific methods and assumptions used for the CHD mortality BAPC model in England and Wales. Text S2, Mean absolute percent error. Description of the type of error measurement used to compare scenarios and models. Text S3, References. References used in the Supplementary information section. (DOCX) [file pone.0099482.s001.docx]

# S. Supplementary information

## S1. Bayesian age, period and cohort model

The APC model is often regarded as a log-linear Poisson model. Here we model the logit of the probability of death from CHD in age group *i* in period *j* as a linear combination of an intercept $\mu$, age effects$\theta_{i }\left(i=1,\ldots I\right)$, period effects and cohort effects$\psi_{k}\left(k=1,\ldots K\right)$:

$I$ is the total number of age groups and $J$the total number of periods. Cohorts are defined by $k=C\left(I-i\right)+j$ and the total number of cohorts is $K=C\left(I-1\right)+J$, where $C$ is the width of the age bands.

To make the model identifiable, the so called “sum to zero” constraints need to be added. The model is only identifiable when it is possible to obtain a unique set of parameters after infinite linear transformations. There is another identifiability problem due to the collinear relationship of age, period and cohort effects which makes it impossible to identify and interpret the separate contributions of each individual effect [1]. However, non-linear trends or change points can be interpreted because the non-identifiability only affects linear trends [2].

### S1A. Random walk of first and second order

For the Bayesian APC model, it is assumed that effects from adjacent time bands tend to be more alike [3]. There are two types of priors that can represent such relationship: a first difference prior and a second difference prior.

In a first difference prior, each effect is derived from the immediately preceding effect, thus preventing it from varying much with respect to adjacent estimates. Additionally, a restriction is added to bound those first differences stochastically to zero and preserve a constant trend. This is known as a random walk of first order (RW1) and for the age effect is described by:

$\theta_{i}\sim N\left(\theta_{i-1},\kappa^{-1}\right), i=2,\ldots,I$.

$$p(\theta_{1})\propto const.$$

where the hyperparameter $\kappa$ is also known as the precision parameter: larger values allow baseline effects to vary only slightly, while small values allow more heavy variation. This model will be the equivalent to scenario A where constant trends are assumed and it also was the type of BAPC model used by Huovinen et al. [4] in their projections for the Finnish population (see section 4.3).

In a second difference prior, each effect is derived from its two immediate predecessors. A random walk of second order (RW2) restricts the second differences stochastically to zero, which penalizes deviations from a linear trend:

$\theta_{i}\sim N\left(2\theta_{i-1}-\theta_{i-2},\kappa^{-1}\right), i=2,\ldots,I$.

$$p(\theta_{1})=p(\theta_{2})\propto const.$$

For both types of prior, $\kappa$ is assumed to follow a gamma distribution $G\left(a,b\right).$ For RW1 $a=1$ and $b=0.001$are set as initial values, and for RW2 initial values are $a=1$ and $b=10^{-5}$. Similar priors are given to the period and cohort effects $\psi$ with hyperparameters $\lambda$and $\nu$, respectively.

Because the non-identifiability only affects linear trends, the RW1 solves the problem by keeping the age, period and cohort effects as constant as possible. However, the RW2 parameters are unidentifiable since the RW2 assumes a linear time trend. Fortunately in a Bayesian framework, it is not essential to ensure identifiability of age, period and cohort effects because $log\left(\frac{p_{ij}}{1-p_{ij}}\right)$ is fully identified [5]. Additionally, many have considered the problem of identifiability in the APC context as unsolvable [6-8].

Finally, the model can be easily extended to account for additional unstructured heterogeneity which cannot be explained by the age, period or cohort effects but by unknown and unobserved covariates:

.

$$z_{ij}\sim N\left(0,\delta^{-1}\right),$$

where the hyperparameter $\delta$ is assumed to follow a gamma distribution$G\left(a_{\delta},b_{\delta}\right)$ with the same starting values as $\kappa.$

### S1B. Estimation, prediction and comparison

The models can be implemented in BAMP [2] which uses Markov chain Monte Carlo (MCMC) simulation for the model estimation. MCMC constructs a Markov chain whose stationary distribution approximates the posterior distribution of interest using a single-component Metropolis-Hastings algorithm [9] for the age, period and cohort effects and a Gibbs sampler algorithm [10] for the hyperparameters.

After it has converged to its stationary distribution, samples from the chain can be used to calculate the parameter estimates and credible intervals from the posterior distribution. A $100\left(1-2p\right)\%$credible interval $\left[c_{p}-c_{1-p}\right]$is a Bayesian interval estimate where $c_{p }$is equal to the $p^{th}$quantile of the scalar component and where $c_{1-p }$is equal to the $\left(1-p\right)^{th}$quantile. Their interpretation differs from the frequentist confidence interval. For example a 95% CI of (18.32%- 67.05%) indicates that the posterior probability that the real percentage change lies between 18.32% and 67.05% is 95%. In this regard, credible intervals capture the current uncertainty about the location of a parameter value and thus can be interpreted as probabilistic statement about the parameter. In contrast, confidence intervals capture the uncertainty about the obtained interval (i.e. whether it contains the true value or not). Thus, they cannot be interpreted as a probabilistic statement about the true parameter values.

Future rates $p_{ij}, j=J+1,\ldots j+T$ can be calculated by projecting the age, period and cohort effects into the future by repeated application of the RW1 or RW2 model definitions. Similarly projected effects $z_{ij}, j=J+1,\ldots j+T$ can be computed independently from$N\left(0,\delta^{-1}\right)$.

Finally, when different BAPC models need to be compared (e.g. RW1 versus RW2), the Deviation Information Criterion (DIC) [11] provides a simple yet powerful way of evaluating different models. DIC is the sum of two components: a term that measures goodness of fit known as posterior expectation of the deviance and a penalty term for increasing model complexity. Models with smaller DIC should be preferred.

### S1C. BAPC model for CHD mortality in England and Wales

For scenario B we assumed that mortality trends will continue, therefore a model with RW2 priors, which assumes linear time trends, seems to be more appropriate. We fitted two RW2 models to our data: one with the unstructured heterogeneity parameter and one without it. We then selected the model with the lowest value of DIC to calculate future probabilities of death from CHD.

The software BAMP uses the number of CHD deaths $y_{ij}$ and start-of-the-year population estimates $l_{ij}$as inputs. However the ONS publishes mid-year population estimates (and projections) only. So to calculate start-of-the-year population estimates, we used the following relationship:

$$l_{ij}=L_{ij}+\frac{y_{ij}}{2}$$

where $L_{ij}$ is the mid-year population estimates.

Notice the other methods presented in this manuscript (Lee-Carter and standardisation of rates) use mortality rates $m_{ij}$, whereas the BAPC model uses mortality probabilities $p_{ij}$,. To convert $p_{ij}$ into $m_{ij}$ so all methods were comparable, we used the following equation [12]:

$$m_{ij}=\frac{p_{ij}}{1-\frac{p_{ij}}{2}}$$

Finally, we were also interested in evaluating the effects of age, period and cohort components in CHD mortality (see section 2.1.1). However, due to the non-identifiably described above, the parameters of a RW2 model cannot be interpreted. Therefore, we also estimated a RW1 model with unstructured heterogeneity parameter in order to evaluate the age, period and cohort component contributions. Although, a RW1 model works under different assumptions, it should not be very different from a RW2 model when describing past trends (i.e. interpolation), giving us a reasonable idea of the size of the effects for the age, period and cohort components.

The hyperparameter estimates are described in Table A.1. Larger values allow baseline effects to vary only slightly, while small values allow more heavy variation, therefore one can see that the age effect explained most variation, followed by the period effect, and then the cohort effect. See section 4.4 for a discussion of these results.

| Hyperparameter | Men | Women |
| --- | --- | --- |
| Age $\left(\kappa\right)$ | 1.6 | 1.0 |
| Period $\left(\lambda\right)$ | 643.5 | 564.8 |
| Cohort $\left(\nu\right)$ | 1303.5 | 868.2 |

Table S.1: Hyperparameter estimates calculated by the RW1 + heterogeneity model.

## S2. Mean absolute percent error

Let $f_{ij}$be the forecast value and $y_{ij}$be the actual observation in age group $i$ and period$j$*.* The *relative* *error* is defined as

$$RE_{ij}=\frac{y_{ij}-f_{ij}}{y_{ij}}$$

The *mean absolute percent error* (*MAPE*) for age group *i* across J periods is:

$$MAPE_{i}=\left(\frac{1}{J}\sum_{j=1}^{J} |RE_{ij}|\right)\times100$$

## S3. References

1. Holford T (2005) Age-period-cohort analysis. In: Armitage P, Colton T, editors. Encyclopedia of Biostatistics. 2nd ed. West Sussex: John Wiley and Sons. pp. 105-123.

2. Schmid VJ, Held L (2007) Bayesian age-period-cohort modeling and prediction-BAMP. J Stat Software 21: 1-15.

3. Clayton D (1996) Generalized Linear Mixed Models. In: Gilks WR, Richardson S, Spiegelhalter DJ, editors. Markov chain Monte Carlo in practice. London: Chapman & Hall. pp. 274-301.

4. Huovinen E, Härkänen T, Martelin T, Koskinen S, Aromaa A (2006) Predicting coronary heart disease mortality—assessing uncertainties in population forecasts and death probabilities by using Bayesian inference. International journal of epidemiology 35: 1246-1252.

5. Knorr-Held L, Rainer E (2001) Projections of lung cancer mortality in West Germany: a case study in Bayesian prediction. Biostatistics 2: 109-129.

6. Glenn ND (1976) Cohort analysts' futile quest: Statistical attempts to separate age, period and cohort effects. American sociological review 41: 900-904.

7. Goldstein H (1979) Age, period and cohort effects: A confounded confusion. Journal of Applied Statistics 6: 19-24.

8. Glenn ND (2005) Cohort analysis: SAGE Publications, Incorporated.

9. Metropolis N, Rosenbluth A, Rosenbluth M, Teller A, Teller E (1953) Equation of state calculations by fast computing machines. The Journal of chemical physics 21: 1087.

10. Geman S, Geman D (1984) Stochastic relaxation, Gibbs distributions, and the Bayesian restoration of images. Pattern Analysis and Machine Intelligence, IEEE Transactions on: 721-741.

11. Spiegelhalter DJ, Best NG, Carlin BP, Van Der Linde A (2002) Bayesian measures of model complexity and fit. Journal of the Royal Statistical Society: Series B (Statistical Methodology) 64: 583-639.

12. Preston S, Heuveline P, Guillot M (2001) Demography: Measuring and modeling population processes. Oxford: Blackwell.
